# Supplementary material for: Mapping the brain atrophy mediating increased impatience for reward in frontotemporal dementia
Source: Commun Biol. 2026 Jan 9;9:172. doi: 10.1038/s42003-025-09450-5 (PMC12876987; doi:10.1038/s42003-025-09450-5)
Supplement: Supplementary file 2 — Supplementary Information [file 42003_2025_9450_MOESM2_ESM.pdf]

SUPPLEMENTARY MATERIALS

Supplementary Figure 1. Effect of bvFTD on discount rate (after removing incoherent participants). 2

Supplementary Figure 2. Correlations between residual discount rates and residual sensitivities to larger later reward for money and food. 3

Supplementary Figure 3. Neuroanatomical whole-brain mediators for the effect of bvFTD on discount rate and on sensitivity to larger later reward for monetary rewards. 4

Supplementary Table 1. Combination of SS, LL amounts, and delays used in the two delay discounting paradigms with money and food stimuli. 5

Supplementary Table 2. Correlations between intertemporal choice task outcomes (discount rate and sensitivity to LL reward) and measures of bvFTD symptoms. 6

Supplementary Table 3. Detailed results of whole-brain mediation analyses with discount rates (log(k)) for money rewards. 7

Supplementary Table 4. Detailed results of whole-brain mediation analyses with sensitivity to LL reward for money rewards. 9

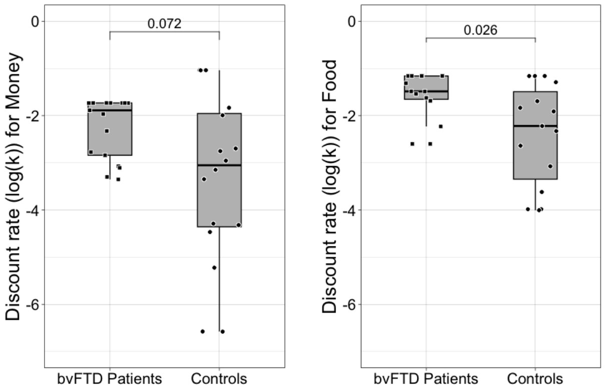

Supplementary Figure 1. Effect of bvFTD on discount rate after removing incoherent participants.

On the left: Wilcoxon test of the difference between bvFTD patients (N=17) and controls (N=16) on discount rate for Money after removing the 5 bvFTD patients showing incoherent intertemporal choices with Money. On the right: Wilcoxon test of the difference between bvFTD patients (N=15) and controls (N=15) on discount rate for Food after removing the 6 bvFTD patients showing incoherent intertemporal choices with Food. Results remain globally similar after removing incoherent participants, i.e., discount rates are higher in bvFTD patients compared to controls (with a close-to-significant effect for Money and a significant effect for Food).

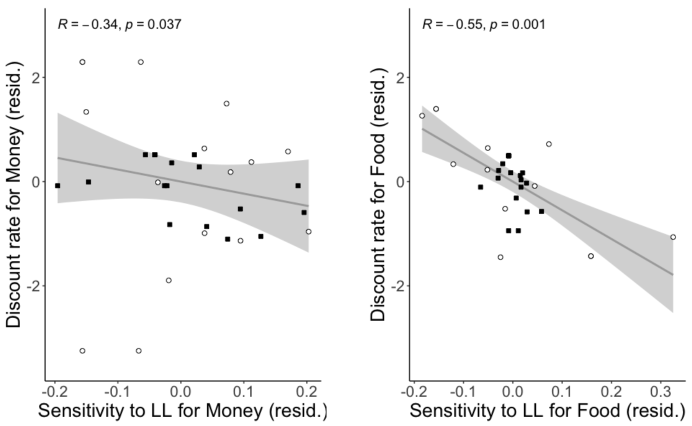

Supplementary Figure 2. Correlations between residual discount rates and residual sensitivities to larger later reward for money and food.

On the left: Spearman correlation between the residual discount rate (independent of group effect) for Money and the residual sensitivity to larger later reward (independent of group effect) for Money across bvFTD patients (N=22; represented as black squares) and controls (N=16; represented as white circles). On the right: Spearman correlation between the residual discount rate (independent of group effect) for Food and the sensitivity to larger later reward (independent of group effect) for Food across bvFTD patients (N=20 after removing one extreme outlier on sensitivity to larger later reward; represented as black squares) and controls (N=13 after removing two extreme outliers on sensitivity to larger later reward and two extreme outliers on discount rate; represented as white circles).

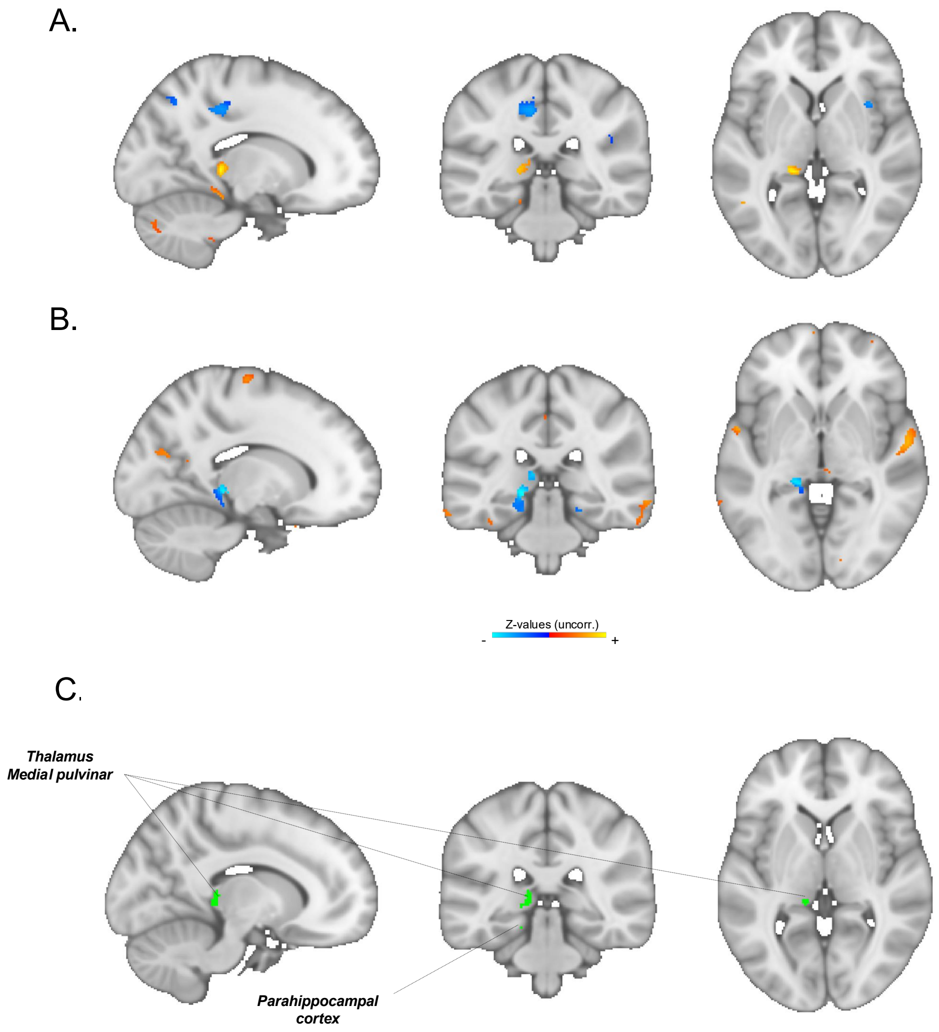

Supplementary Figure 3. Neuroanatomical whole-brain mediators for the effect of bvFTD on discount rate and on sensitivity to larger later reward for monetary rewards.
A: *Path ab* of whole-brain mediation, which represents brain regions in which grey matter density mediates the effect of bvFTD (n=22) vs controls (n=16) on discount rate (for money), after masking with the map of the intersection between *Path a*, *Path b* and *Path ab*. Voxels in orange correspond to the positive mediators that contribute to the increase in discounting due to bvFTD. Only uncorrected results (thresholded at p<0.05) in orange and light blue are displayed.
B: *Path ab* of whole-brain mediation, which represents brain regions in which grey matter density mediates the effect of bvFTD (n=22) vs controls (n=16) on sensitivity to larger later reward (for money), after masking with the map of the intersection between *Path a*, *Path b* and *Path ab*. Voxels in blue correspond to the negative mediators that contribute to the decrease in sensitivity to larger later reward due to bvFTD. Only uncorrected results (thresholded at p<0.05) in orange and light blue are displayed.
C: Intersection of the two intersection maps between *Path a*, *Path b* and *Path ab* for the whole-brain mediations of the effect of bvFTD on discount rate on the one hand and on sensitivity to larger later reward on the other hand. Overlapping mediator clusters are located mostly in the medial pulvinar and to a lesser extent in the parahippocampal cortex.

Supplementary Table 1. Combination of SS, LL amounts, and delays used in the two delay discounting paradigms with money and food stimuli.
The presentation order was randomized for each participant. Indifference k denotes the discounting rate at which the SS and LL options should be chosen at equal proportions.

SS delay (in days)	LL delay (in days)	SS amount (in euros or chocolates)	LL amount (in euros or chocolates)	Indifference k
0	14	8	10	0.017857143
0	14	8	32	0.214285714
0	14	17.5	20.5	0.012244898
0	14	21	21.25	0.00085034
0	14	22.5	32.5	0.031746032
0	14	23	25.75	0.008540373
0	14	23.5	23.7	0.000607903
0	14	27	36	0.023809524
0	14	28	56	0.071428571
0	28	12	13.5	0.004464286
0	28	12	24	0.035714286
0	28	14	14.2	0.000510204
0	28	15	20	0.011904762
0	28	16	23	0.015625
0	28	17.1	17.2	0.000208855
0	28	21	24.5	0.005952381
0	28	24	96	0.107142857
0	28	31	39	0.00921659
14	28	10.15	10.3	0.001071429
14	28	15.75	17.5	0.008928571
14	28	17	24	0.05
14	28	18	24	0.035714286
14	28	21.1	21.2	0.000340136
14	28	28	33	0.01552795
14	28	35	44	0.024725275
28	42	9	10.5	0.017857143
28	42	11	13.75	0.035714286
28	42	14.05	14.1	0.000256016
28	42	22.25	22.65	0.001332001
28	42	24	32	0.071428571
28	42	26	29	0.010714286
28	42	28	40	0.214285714

Supplementary Table 2. Correlations between intertemporal choice task outcomes (discount rate and sensitivity to LL reward) and measures of bvFTD symptoms.
Spearman correlations are calculated among bvFTD patients (N=24). Hayling (errors): objective measure of inhibition deficit from the Hayling Sentence Completion Test (number of errors in the inhibition phase of the test); FAB: Frontal Assessment Battery, global measure of frontal executive functioning; EBI: Eating Behavior Inventory, global measure of changes in eating behaviour. DAS-Exe: Dimensional Apathy Scale, Executive subscale measuring lack of executive functions (e.g., lack of planning) to complete goal-directed behaviors.

	**Hayling (errors)**	**FAB**	**EBI**	**DAS-Exe**
Discount rate for money	0.67[a]	-0.41[c]	-0.004	0.20
Discount rate for food	0.21	0.02	0.15	0.29
Sensitivity to LL reward for money	-0.41[c]	0.57[b]	0.006	-0.26
Sensitivity to LL reward for food	-0.09	-0.05	-0.14	-0.005

[a] Significant correlation at $p < 0.001$
[b] Significant correlation at $p < 0.01$
[c] Close-to-significant correlation at $p < 0.1$

Supplementary Table 3. Detailed results of whole-brain mediation analyses with discount rates (log(k)) for money rewards.
For paths b and ab, we list all the clusters significant at q<0.05 (= p<0.008) FDR-corrected (across paths a, b and ab) corresponding to both positive and negative effects. Atlas label: reference region with highest number of in-region voxels. Volume: volume of contiguous region in cubic mm. X, Y and Z: peak coordinates in MNI space. Max(z): signed max over p. Clusters in bold correspond to cortical and subcortical regions of overlap between paths a (negative effects), b (negative effects) and ab (positive effects).

Whole-brain mediation: Discount rate for money – Path b

Positive effects

Name	Atlas label	Volume	X	Y	Z	max(z)
Precuneous cortex	Ctx_31pd_R	56	12	-53	32	7.0345
Inferior frontal gyrus	Ctx_45_L	80	-53	27	-2	7.0345
Lateral occipital cortex	Ctx_MIP_L	504	-18	-66	48	7.0345
Supramarginal gyrus	Ctx_PFt_R	1016	53	-36	51	7.0345
Precuneous cortex	Ctx_7Am_R	264	8	-57	65	7.0345
Precuneous cortex	Ctx_POS2_R	848	5	-72	44	7.0345
Ventromedial prefrontal cortex	Ctx_10v_L	136	-3	27	-24	7.0345
Precentral gyrus	Ctx_4_L	376	-6	-30	53	7.0345
Precuneous cortex	Ctx_23c_L	976	-14	-36	42	7.0345
Occipital fusiform gyrus	Ctx_V4_R	256	26	-78	-6	7.0345
Occipital pole	Ctx_V1_R	392	21	-98	-2	6.7649
Precuneous cortex	Ctx_7Pm_R	64	2	-63	53	6.7551
Middle frontal gyrus	Ctx_8C_L	40	-38	21	30	6.4902
Angular gyrus	Ctx_7PC_L	112	-45	-54	53	6.0388
Precuneous cortex	Ctx_PCV_L	96	-8	-53	53	4.0588
Occipital pole	Ctx_V3_L	96	-26	-95	3	3.6701

Negative effects

Name	Atlas label	Volume	X	Y	Z	max(z)
Cerebellum	Cblm_VI_L	976	-29	-39	-33	-22.125
Cerebellum	Cblm_VIIIa_R	4936	11	-72	-45	-17.87
Cerebellum	Cblm_Vermis_VI	576	-2	-74	-14	-15.356
Cerebellum	Cblm_I_IV_L	64	-11	-44	-5	-14.873
**Thalamus pulvinar**	**Thal_Pulv**	**256**	**-14**	**-35**	**3**	**-14.581**
**Parahippocampal cortex**	**Ctx_PHA1_L**	**168**	**-17**	**-38**	**-14**	**-13.811**
**Middle temporal gyrus**	**Ctx_STV_R**	**240**	**65**	**-47**	**11**	**-12.806**
Inferior temporal gyrus	Ctx_TE2p_L	32	-50	-42	-15	-12.562
Cerebellum	Cblm_VIIIa_L	704	-12	-69	-51	-10.549
Cerebellum	Cblm_CrusI_L	984	-24	-83	-27	-8.4412

Cerebellum	Cblm_IX_R	48	8	-53	-35	-7.71
Angular gyrus	Ctx_IP2_R	272	41	-50	47	-7.192
Cerebellum	Cblm_CrusII_L	464	-33	-72	-42	-6.4424
Occipital pole	Ctx_V1_L	96	-11	-93	11	-6.2466
Lateral occipital cortex	Ctx_FST_R	104	45	-69	-6	-6.1674
Lateral occipital cortex	Ctx_MT_L	48	-44	-72	9	-5.2595
Occipital fusiform gyrus	Ctx_V3_L	88	-18	-71	-9	-5.2073
**Occipital fusiform gyrus**	**Ctx_V8_L**	**112**	**-35**	**-71**	**-14**	**-4.0465**
Angular gyrus	Ctx_PGi_R	488	50	-56	26	-3.6751

Whole-brain mediation: Discount rate for money – Path ab

Positive effects

**Name**	**Atlas label**	**Volume**	**X**	**Y**	**Z**	**max(z)**
**Thalamus pulvinar**	**Thal_Pulv**	**296**	**-14**	**-33**	**3**	**0.98686**
Superior temporal gyrus	Ctx_STSdp_L	192	-56	-23	-3	0.89737
Middle temporal gyrus	Ctx_TPOJ2_L	384	-48	-57	5	0.85809
Middle frontal gyrus	Ctx_9_46d_L	424	-23	36	29	0.75078
Middle temporal gyrus	Ctx_TPOJ1_L	104	-66	-42	3	0.7085
Lateral occipital cortex	Ctx_TPOJ2_L	80	-51	-66	9	0.69456
**Middle temporal gyrus**	**Ctx_STV_R**	**672**	**60**	**-45**	**8**	**0.68241**
Parahippocampal gyrus	No label	80	-15	2	-29	0.65357
Precentral gyrus	Ctx_6a_R	152	26	-11	54	0.64242
**Parahippocampal gyrus**	**Ctx_PHA1_L**	**384**	**-15**	**-38**	**-12**	**0.61398**
Cerebellum	Cblm_CrusI_L	160	-24	-83	-24	0.59469
Cerebellum	Cblm_Vermis_VI	272	-3	-74	-9	0.55765
Inferior temporal gyrus	Ctx_TE2p_L	80	-48	-41	-15	0.55005
Insular cortex	Ctx_MI_R	88	44	15	-3	0.51757
Cerebellum	Cblm_Vermis_VI	40	2	-72	-12	0.47083
Lateral occipital cortex	Ctx_V3CD_R	64	30	-78	12	0.46013
Cerebellum	Cblm_CrusI_L	112	-20	-81	-33	0.42299
**Occipital fusiform gyrus**	**Ctx_V8_L**	**64**	**-33**	**-69**	**-14**	**0.38635**

Negative effects

**Name**	**Atlas label**	**Volume**	**X**	**Y**	**Z**	**max(z)**
Brain stem	Ctx_23c_L	456	-12	-36	42	-0.66327
Lateral occipital cortex	Ctx_MIP_L	264	-18	-66	50	-0.48952
Parietal operculum cortex	Ctx_PFcm_R	280	44	-33	23	-0.29149

Supplementary Table 4. Detailed results of whole-brain mediation analyses with sensitivity to LL reward for money rewards.
For paths b and ab, we list all the clusters significant at q<0.05 (= p<0.008) FDR-corrected (across paths a, b and ab) corresponding to both positive and negative effects. Atlas label: reference region with highest number of in-region voxels. Volume: volume of contiguous region in cubic mm. X, Y and Z: peak coordinates in MNI space. Max(z): signed max over p. Clusters in bold correspond to cortical and subcortical regions of overlap between paths a (negative effects), b (positive effects) and ab (negative effects).

Whole-brain mediation: Sensitivity to LL reward for money – Path b

Positive effects

Name	Atlas label	Volume	X	Y	Z	max(z)
**Parahippocampal gyrus**	**'Ctx_PreS_L'**	**816**	**-17**	**-33**	**-8**	**1.6773**
**Hippocampus**	**'Ctx_PHA1_R'**	**176**	**20**	**-27**	**-18**	**1.5495**
Lateral Occipital Cortex	'Ctx_MST_L'	64	-41	-65	2	1.1964
Lateral Occipital Cortex	'Ctx_PGp_R'	912	36	-83	32	1.0119
Lateral Occipital Cortex	'Ctx_TPOJ1_R'	80	47	-38	9	0.97698
Lateral Occipital Cortex	'Ctx_V3CD_R'	48	32	-81	8	0.88435
Lateral Occipital Cortex	'Ctx_FST_R'	432	41	-62	0	0.72675
Occipital Fusiform Gyrus	'Ctx_V3_L'	128	-20	-86	-8	0.55461
**Superior Frontal Gyrus**	**'Ctx_8Ad_L'**	**176**	**-23**	**21**	**47**	**0.44211**

Negative effects

Name	Atlas label	Volume	X	Y	Z	max(z)
Cerebellum	'Cblm_I_IV_L'	15880	-3	-47	-17	-2.0571
Inferior Temporal Gyrus	'Ctx_FFC_L'	152	-44	-42	-29	-1.2322
Cerebellum	'Cblm_CrusI_R'	792	51	-60	-41	-1.2258
Inferior Frontal Gyrus	'Ctx_IFSp_L'	184	-54	27	17	-1.0819
Temporal Pole	'No label'	32	-56	2	-41	-1.0679
Temporal Fusiform Cortex	'Ctx_TF_R'	288	38	-11	-29	-1.0528
Temporal Pole	'Ctx_TE1a_L'	112	-60	3	-26	-0.98935
Middle Temporal Gyrus	'Ctx_TE1p_L'	200	-68	-39	-12	-0.98538
Precentral Gyrus	'Ctx_6mp_R'	2304	11	-14	69	-0.96885

Middle Frontal Gyrus	'Ctx_i6_8_R'	1368	36	15	54	-0.96489
Cerebellum	'Cblm_Vermis _CrusII'	64	3	-77	-29	-0.95939
Middle Temporal Gyrus	'Ctx_TE1p_R'	2776	68	-33	-14	-0.95659
Lingual Gyrus	'Ctx_V4_R'	160	18	-66	-3	-0.93646
Superior Temporal Gyrus	'Ctx_A5_R'	1792	63	-8	-3	-0.91319
Lateral Occipital Cortex	'Ctx_V6A_R'	224	20	-83	45	-0.90264
Middle Temporal Gyrus	'Ctx_TE1a_L'	216	-65	-15	-29	-0.88901
Supramarginal Gyrus	'Ctx_PFt_R'	344	50	-35	42	-0.88724
Inferior Temporal Gyrus	'Ctx_TE2a_R'	160	62	-18	-33	-0.88707
Intracalcarine Cortex	'Ctx_V1_L'	120	-14	-83	2	-0.88039
Cerebellum	'Cblm_CrusI_L'	240	-41	-51	-24	-0.86183
Middle Temporal Gyrus	'Ctx_TE1a_R'	112	56	0	-41	-0.85137
Postcentral Gyrus	'Ctx_3b_L'	120	-53	-15	38	-0.8336
Inferior Temporal Gyrus	'Ctx_TF_L'	176	-44	-20	-36	-0.81291
Frontal Pole	'Ctx_a47r_R'	176	36	60	2	-0.81155
Inferior Temporal Gyrus	'Ctx_TGv_R'	48	45	-8	-48	-0.81137
Lateral Occipital Cortex	'Ctx_PFm_L'	256	-33	-65	54	-0.80347
Cerebellum	'Cblm_CrusI_R'	80	50	-62	-30	-0.79548
Occipital Fusiform Gyrus	'Ctx_V4_R'	32	30	-83	-12	-0.77974
Precentral Gyrus	'Ctx_6d_R'	200	29	-12	68	-0.76772
Temporal Occipital Fusiform Cortex	'Ctx_VVC_L'	176	-35	-53	-15	-0.73857
Inferior Temporal Gyrus	'Ctx_TE2a_R'	240	50	-14	-32	-0.73817
Temporal Occipital Fusiform	'Ctx_FFC_R'	392	38	-41	-23	-0.70385
Postcentral Gyrus	'Ctx_PFop_R'	56	66	-17	27	-0.70247
Middle Temporal Gyrus	'Ctx_TE1m_L'	48	-69	-29	-20	-0.6964
Superior Temporal Gyrus	'Ctx_A5_L'	544	-65	-14	-5	-0.69391
Middle Frontal Gyrus	'Ctx_i6_8_R'	512	35	3	62	-0.69047
Precentral Gyrus	'Ctx_55b_L'	176	-51	5	48	-0.68912
Precentral Gyrus	'Ctx_6mp_L'	848	-12	-14	74	-0.66482

Superior Temporal Gyrus	'Ctx_A5_L'	104	-63	-5	-5	-0.64897
Supramarginal Gyrus	'Ctx_PF_L'	584	-65	-36	24	-0.64608
Precuneous Cortex	'Ctx_DVT_R'	64	23	-56	17	-0.64234
Superior Parietal Lobule	'Ctx_7AL_R'	208	23	-56	62	-0.55565
Inferior frontal gyrus	'Ctx_44_R'	176	56	29	18	-0.54649
Middle Frontal Gyrus	'Ctx_8Av_R'	160	33	35	47	-0.54483
Occipital Fusiform Gyrus	'Ctx_V3_R'	128	20	-80	-9	-0.53625
Precentral gyrus	'Ctx_FEF_R'	72	48	-5	51	-0.48117
Inferior Temporal Gyrus	'Ctx_TE2a_R'	360	50	-8	-39	-0.47879
Angular Gyrus	'Ctx_PGi_L'	40	-51	-51	24	-0.47834
Superior Parietal Lobule	'Ctx_2_L'	96	-41	-39	53	-0.43458
Middle Temporal Gyrus	'Ctx_PHT_R'	32	59	-54	6	-0.38895
Occipital Fusiform Gyrus	'Ctx_V3_L'	32	-26	-84	-17	-0.33322

Whole-brain mediation: Sensitivity to LL reward for money – Path ab

Positive effects

Name	Atlas label	Volume	X	Y	Z	max(z)
Planum Polare	'Ctx_TA2_R'	208	50	-9	-8	0.083244
Middle Temporal Gyrus	'Ctx_TE1a_L'	248	-66	-17	-9	0.072078
Inferior Temporal Gyrus	'Ctx_TGd_L'	160	-54	0	-42	0.071929
Temporal Pole	'Ctx_TE1a_L'	208	-60	3	-26	0.071332
Cerebellum	'Cblm_VI_L'	2312	-35	-42	-23	0.067373
Inferior Temporal Gyrus	'Ctx_TE2a_R'	592	53	-12	-29	0.065779
Superior Temporal Gyrus	'Ctx_A5_R'	1008	60	-5	-3	0.0626
Middle Frontal Gyrus	'Ctx_8Av_R'	1328	38	17	51	0.061144
Precentral Gyrus	'Ctx_FEF_R'	48	48	-5	51	0.05866
Temporal Occipital Fusiform Cortex	'Ctx_FFC_R'	712	41	-42	-23	0.0579
Middle Temporal Gyrus	'Ctx_TE1a_L'	344	-63	-17	-27	0.057327

Inferior Temporal Gyrus	'Ctx_TE1a_R'	184	56	-2	-39	0.05726
Cerebellum	'Cblm_V_R'	984	18	-51	-12	0.056781
Middle Temporal Gyrus	'Ctx_TE1m_R'	1872	66	-30	-12	0.052876
Occipital Fusiform Gyrus	'Ctx_V3_R'	480	20	-84	-9	0.051847
Lateral Occipital Cortex	'Ctx_V6A_R'	272	20	-83	45	0.051538
Inferior Temporal Gyrus	'Ctx_TE2a_R'	408	62	-18	-33	0.050419
Middle Frontal Gyrus	'Ctx_i6_8_R'	160	35	0	60	0.048325
Middle Temporal Gyrus	'Ctx_TE1a_R'	496	60	5	-27	0.048232
Cuneal Cortex	'Ctx_V2_L'	144	-12	-74	21	0.047721
Precentral Gyrus	'Ctx_6mp_L'	256	-15	-17	71	0.045836
Middle Temporal Gyrus	'Ctx_TE1p_L'	136	-68	-38	-14	0.045816
Insular Cortex	'Ctx_AAIC_R'	24	42	14	-11	0.045439
Inferior Temporal Gyrus	'Ctx_TF_L'	208	-45	-18	-38	0.045346
Frontal Pole	'Ctx_a47r_R'	192	35	60	3	0.044765
Middle Frontal Gyrus	'Ctx_8C_L'	144	-39	18	39	0.044342
Planum Temporale	'Ctx_A4_L'	80	-63	-12	3	0.043938
Precentral Gyrus	'Ctx_6mp_R'	64	15	-14	75	0.043554
Inferior Temporal Gyrus	'Ctx_TE2a_R'	384	48	-6	-39	0.043398
Subcallosal Cortex	'Cau_R'	32	3	26	-5	0.042432
Temporal Fusiform Cortex	'Ctx_TF_R'	144	38	-6	-32	0.04224
Superior Parietal Lobule	'Ctx_7AL_R'	168	20	-56	60	0.041508
Middle Frontal Gyrus	'No label'	80	50	18	39	0.039425
Inferior Temporal Gyrus	'Ctx_TGv_R'	344	44	-8	-47	0.038595
Middle Temporal Gyrus	'Ctx_TE1p_R'	112	68	-38	2	0.03611
Middle Frontal Gyrus	'Ctx_55b_L'	168	-47	5	50	0.036047
Superior Frontal Gyrus	'Ctx_6mp_L'	64	-9	-8	74	0.034811
Intracalcarine Cortex	'Ctx_V1_L'	72	-11	-83	2	0.032736
Cerebellum	'Cblm_VI_L'	64	-24	-59	-15	0.028397

Negative effects

Names	Atlas label	Volume	X	Y	Z	max(z)
**Parahippocampal Gyrus**	**'Ctx_PHA1_R'**	**128**	**20**	**-27**	**-20**	**-0.096301**
**Hippocampus**	**'Ctx_PreS_L'**	**528**	**-17**	**-33**	**-6**	**-0.088125**
Cerebellum	'Cblm_I_IV_L'	288	-3	-45	-8	-0.060227
**Superior Frontal Gyrus**	**'Ctx_8Ad_L'**	**216**	**-21**	**21**	**48**	**-0.058439**
